# Impact of short chain fatty acids (SCFAs) on antimicrobial activity of new β-lactam/β-lactamase inhibitor combinations and on virulence of *Escherichia coli* isolates

**DOI:** 10.1038/s41429-023-00595-1

**Published:** 2023-02-01

**Authors:** Ashraf A. Kadry, May A. El-Antrawy, Amira M. El-Ganiny

**Affiliations:** 1 Microbiology and Immunology Department, Faculty of Pharmacy, Zagazig University, Zagazig, 44519 Egypt; 2 Microbiology and Biotechnology Department, Faculty of Pharmacy, Delta University for Science and Technology, Gamasa, 11152 Egypt

**Keywords:** Bacteriology, Antibiotics, Bacteria

## Abstract

In a healthy gut microbiota, short chain fatty acids (SCFAs) are produced. The antibacterial action of SCFAs against intestinal pathogens makes them useful for ensuring the safety of food and human health. In this study, we aimed to assess the in vitro inhibitory activity of SCFAs, and to report, for the first time, their impact on the activity of new β-lactam/β-lactamase inhibitor combinations. The minimum inhibitory concentrations of acetic, propionic, and butyric acids were determined against *E. coli* clinical isolates recovered from gastrointestinal infections. Cefoperazone/sulbactam, ceftazidime/avibactam and cefepime/enmetazobactam are new β-lactam/β-lactamase inhibitor combinations that were studied for their combined therapeutic effects. Also, the effects of pH and concentration of SCFAs were evaluated on in vitro bacterial growth and expression of genes encoding for motility, adhesion, invasion, and biofilm formation. SCFAs were tested at concentrations of 12 mM at pH 7.4 (ileum-conditions), in addition to 60 mM and 123 mM, at pH 6.5 (colon-conditions). The tested SCFAs showed the same MIC (3750 μg ml^−1^ ≃ 60 mM) against all isolates. Furthermore, the addition of SCFAs to the tested β-lactam/β-lactamase inhibitor combinations greatly restored the susceptibility of the isolates. SCFAs had significant effect on bacterial growth and virulence in a pH and concentration-dependent manner; low ileal concentration potentiated *E. coli* growth, while higher colonic concentration significantly suppressed growth and down-regulated the expression of virulence genes (*fliC, ipaH, FimH, BssS*). Therefore, the significant inhibitory effect of colonic SCFAs on β-lactam/β-lactamase inhibitor combinations might lead to the development of promising treatment strategies.

## Key points

- β-lactamase inhibitors restore the susceptibility of *E. coli* isolates.
- SCFAs had inhibitory effect on *E. coli* growth and virulence from GIT infections.
- Effect of SCFAs on the activity of β-lactam/β-lactamase combinations of interest.

## Introduction

*Escherichia coli* is an important member of family *Enterobacteriaceae* that is responsible for many intestinal and extra-intestinal infections in human [1]. *E. coli* strains have an arsenal of virulence factors, which allows them to cause a broad spectrum of infections including urinary tract infections, gastroenteritis, pneumonia, septicemia, and meningitis [2]. Virulence factors are produced by pathogenic bacteria and are encoded by specific genes located on chromosomes or mobile genetic elements [3]. Bacterial cell surface proteins and secreted factors make up the two categories of *E. coli’*s virulence factors. Cell surface virulence factors most commonly include fimbriae that help in adhesion to host cell surface, tissue invasion, biofilm formation and cytokine induction. In addition, cell surface virulence factors include flagella which are responsible of motility. A couple of examples of secreted virulence factors are hemolysin and siderophores [4, 5].

*E. coli* infections can be associated with high mortality rates if not treated properly [6]. Beta-lactam antibiotics such as cephalosporins are the most widely used antimicrobials for treating *E. coli* infections [7]. However, the extensive use of antibiotics has promoted the rapid development of multidrug resistant (MDR) isolates, which have become a global health problem especially in developing countries [8]. β-lactamase enzymes are the major contributors for cephalosporins resistance, for example, extended spectrum β-lactamases (ESBLs) have activity against 3rd and 4th generation cephalosporins in addition to monobactams [9, 10]. Recently, new combinations between cephalosporins and β-lactamase inhibitors have been found to exhibit synergistic activities against MDR organisms, these combinations include cefoperazone/sulbactam, ceftazidime/avibactam and cefepime/enmetazobactam [11, 12]. Ceftazidime/avibactam was approved in the USA in 2015 and in Europe in 2016, cefoperazone/sulbactam is currently approved in some European countries, but not in the USA, while cefepime/enmetazobactam is still to be approved [13]. Although, these new β-lactamase inhibitors were able to enhance the activity of cephalosporins against many clinical MDR *E. coli* isolates in vitro, some of the tested strains remained resistant to such combinations [14]. This points to the need to search for other solutions to such resistance.

Organic acids have a long history of use in the food industry as food preservatives, in particular short chain fatty acids (SCFAs), such as acetic acid (AA), butyric acid (BA), and propionic acid (PA), which are naturally produced by microbiota of the ileum (i-SCFA) and the colon (c-SCFA) [15]. The amount and type of SCFAs produced are influenced by the diet intake, in addition to the composition of the gut microbiota [16]. The amount of total SCFAs in the large intestine ranges from 60 to 123 mM, with acetate, propionate, and butyrate having a molar ratio of 60:20:20, while in the human ileum, lower concentrations of SCFAs (from 7 to 20 mM) have been observed [17]. SCFAs exert several beneficial effects on host metabolism and immune system [18]. They have also been shown to modulate replication, colonization, and virulence of enteric pathogens [19]. For these reasons, SCFAs are of increasing interest for development of new therapeutics.

The aim of current study is to clarify the role of short chain fatty acids (SCFAs) on growth and virulence of *Escherichia coli* clinical isolates recovered from gastrointestinal infections, and to investigate the therapeutic potentials of short chain fatty acids together with the three tested β-lactam/β-lactamase combinations.

## Materials and methods

### Bacterial isolates

In this study, 140 *Escherichia coli* isolates were previously obtained from the laboratory of the Gastrointestinal Surgery Center (GISC) in Mansoura, Egypt. These isolates were identified by standard microbiological methods. The isolates were stored at −20 °C in tryptone soya broth (TSB, Oxoid, UK) with 20% glycerol [20].

### Susceptibility of *E. coli* isolates to β-Lactam/β-Lactamase inhibitor combinations

Three different β-lactam/β-lactamase inhibitor combinations were examined for their antimicrobial activity including cefoperazone/sulbactam, ceftazidime/avibactam, and cefepime/enmetazobactam. Cefoperazone/sulbactam and ceftazidime/avibactam were used at ratios of 1:2, and 4:1, respectively. A fixed concentration of enmetazobactam (8 μg ml^−1^) was used in cefepime/enmetazobactam combination [14]. The Clinical and Laboratory Standards Institute’s broth microdilution method was used to determine minimum inhibitory concentrations (MICs) [21].

Sigma Aldrich (Darmstadt, Germany) provided the test antibiotics: cefoperazone, ceftazidime, and cefepime. MedChemExpress MCE (Monmouth Junction, USA) provided the three β-lactamase inhibitors. All powders were kept at 4 °C in airtight containers. The lowest level of β-lactam antibiotic at which no visible bacterial growth detected was considered as the MIC. Resazurin dye (Sigma-Aldrich, Darmstadt, Germany) was used to detect bacterial growth colorimetrically producing pink, fluorescent resofurin as a byproduct [22].

### Susceptibility of *E. coli* isolates to different SCFAs singly and in combinations

The MICs of acetic acid (Sigma Aldrich, Schnelldorf, Germany), propionic acid and butyric acid (Alfa Aesar, ThermoFisher Scientific, Massachusetts, Waltham, MA, USA) were determined by broth microdilution method according to Clinical and Laboratory Standards Institute [21].

### Susceptibility of *E. coli* isolates for combined therapy of new β-lactam/β-lactamase inhibitor along with SCFAs

The MICs for β-lactam/β-lactamase inhibitor combinations were determined in presence of sub-MIC (½ MIC) of SCFAs at pH 6.5 using the broth microdilution method [21]. Starting with a double strength β-lactam/β-lactamase inhibitor concentration, two-fold serial dilutions were made in microtiter wells leaving one well as growth control and another as negative control. Then ½ the MIC of SCFAs was added (37.5 μl from 10 mg μl^−1^ stock) to each well except for the growth and the negative control (un-inoculated) wells. Finally, 100 μl of bacterial suspension (1.5 × 10^5^ CFU ml^−1^) were added and plates were incubated for 24 h at 37 °C and the MIC was recorded after adding resazurin dye.

### The effect of SCFAs on in vitro bacterial growth of *E. coli*

A 24-h well isolated *E. coli* colony on nutrient agar was used to inoculate 10 ml of MHB that was incubated for overnight at 37 °C. Then, one ml of the incubated culture was used to inoculate 50 ml of sterile MHB in a 100-ml conical flask. The flask was incubated at 37 °C in shaking incubator. One-ml aliquots of the culture were taken for measuring the optical density (OD) at wavelength of 600 nm and for viable counting after 2 h and every 60 min. The viable count defined as the number of colony-forming units (CFU) was determined using the standard plate counting technique [23].

To investigate the effect of SCFAs on bacterial growth, a mixture of acetic, propionic, and butyric acid (with a ratio of 60:20:20 for acetate, propionate, and butyrate; respectively) was added to bacterial suspension of *E. coli* isolates -that remained resistant to β-lactams even after the addition of β-lactamase inhibitors- so as to mimic both ileum and colonic conditions [16]. Similarly, OD was measured after 2 h and every 60 min. At the end, growth curves were drawn for the bacterial isolate in the presence and absence of SCFAs.

### Effect of SCFAs on transcription of virulence genes by quantitative real–time polymerase chain reaction (qRT-PCR)

Fifteen-milliliter falcon tubes were filled with 10 ml MHB and 100 μl of bacterial suspension (10^4^ CFU ml^−1^) from an overnight culture of the test isolate. Then various concentrations of SCFAs were made in the inoculated falcon tubes as follows; i-SCFA (12 mM at pH 7.4) and c-SCFA (60 mM and 123 mM at pH 6.5) or sterile NaCl (Control). The prepared cultures were incubated to mid log phase at 37 °C in orbital shaker then centrifuged at 2000 × *g* for 10 min. The supernatant was discarded, and the pellet was resuspended in 250 μl of TE buffer (10 mM Tris pH 8 and 1 mM EDTA), then 25 μl of 10% Sodium dodecyl sulfate (SDS) was added. The mixture was incubated at 65 °C for 30 min, then 1 ml of trizol was added and the sample was sonicated on ice-bath using probe sonicator (Thermofisher Scientific, CA, USA,) for 2 min [24].

Total RNA was extracted using Qiagen RNA Protect-RNeasy Kit (Qiagen, Hilden, Germany) according to manufacturer’s recommendations. Immediately, RNA concentration and purity was evaluated using Thermo Scientific™ NanoDrop™ One Microvolume UV-Vis Spectrophotometer (Thermofisher Scientific, CA, USA,). RNA was reverse transcribed to cDNA using RevertAid First Strand cDNA Synthesis kit according to manufacture instructions (Biogen, Munich, Germany).

Table 1 demonstrated the primers for the four tested virulence associated genes *fimH*, *ipaH*, *fliC*, and *BssS* that were analyzed using SensiFAST™ SYBR^®^ High-ROX Kit (Bioline USA Inc., USA). The 10 μl total volume of qRT-PCR reaction was made up of 1 μl of cDNA, 0.7 μl of each forward and reverse primers (10 μM), 5 μl of 2× SYBR Green Master Mix, 1 μl of QN ROX Reference Dye and 1.6 μl of nuclease-free water. The amplification conditions were made as follows: initial denaturation at 95 °C for 20 s, followed by 40 cycles of denaturation at 95 °C for 10 s, annealing at 60 °C for 15 s, extension at 72 °C for 30 s, and a final melting curve program of 15 s at 95 °C, 60 s at 60 °C, followed by a dissociation step for 15 s at 95 °C. The reaction was carried out in StepOne™ Real-Time PCR System (Applied Biosystems™, ThermoFisher Scientific, Foster, CA, USA). The comparative quantification method (∆Ct) was used to determine the up-and down-regulated genes, where the expression level of targeted genes in comparison to the control 16 S rRNA gene (Sigma Co., USA) was calculated by using the equation RQ = 2^-∆∆CT^ where ∆∆Ct = (Ct_target genes_ – Ct_16srRNA_)_treatment _− (Ct_target genes_ − Ct_16srRNA_)_control_, where Ct is the threshold cycle.

**Table 1:** Primers used in amplification of targeted virulence genes in Real-Time PCR (Sigma Co., USA)

Gene Name	Gene function	Sequence 5`-3`	Reference
***fimH-F***	**Adhesion**	**CTTATGGCGGCGTGTTATCT**	[45]
***fimH-R***		**CTGCTCACAGGCGTCAAATA**	
***fliC-F***	**Motility**	**ACAGCCTCTCGCTGATCACTCAAA**	[46]
***fliC-R***		**GCGCTGTTAATACGCAAGCCAGAA**	
***ipaH-F***	**Invasion**	**TTGACCGCCTTTCCGATACC**	[47]
***ipaH-R***		**ATCCGCATCACCGCTCAGAC**	
***BssS-F***	**Biofilm formation**	**TCCTGCTCGGACTTATTTGG**	[47]
***BssS-R***		**ATTCAGACTCATCCGCTCGT**	

### The effect of SCFAs on *E. coli* motility

The effect of SCFAs on the swarming motility of *E. coli* isolates was carried out in semisolid agar plates. *E. coli* isolates were grown in Luria-Bertani (LB) broth overnight at 37 °C. Soft agar plates (1% tryptone, 0.5% NaCl, 0.25% agar) were prepared the day before assay. The control plates had the same quantity of saline as the total SCFAs, and SCFAs were added to the agar medium immediately before pouring into the plate. Three μl of *E. coli* overnight cultures were added to the center of each plate. Finally, plates were incubated at 37 °C for 10 h. The experiment was carried out in triplicate [16].

### The Effect of SCFAs on biofilm formation of *E. coli* isolates

The biofilm formation was performed as described by Salo et al. [25]. Briefly, 5 ml of LB broth was inoculated with a test isolate colony and incubated at 37 °C for 18–20 h. Bacterial cells were collected by centrifugation at 5000 rpm for 5 min, the cells were washed with 0.5 ml phosphate buffered saline, followed by centrifugation. The washed cells were resuspended in 1 ml of LB broth and the cell density was adjusted to 10^7^ cells ml^−1^. 200 μl of each test isolate were placed into the round bottomed wells of 96-well polystyrene microtiter plate. SCFAs were added to the test isolates at concentrations of 60 mM and 123 mM and plates were incubated at 37 °C for 24 h. The bacterial suspensions were gently aspirated from the wells that were washed three times each with 200 μl of sterile PBS to remove planktonic cells. The plates were then dried for 45 min at room temperature and 150 μl of 0.4% crystal violet (CV) was added to each well and left for 45 min at room temperature. After this, 150 μl of 95% ethanol was added to each well and left for 45 min. Then, 100 μl of each well was transferred to a fresh 96-well microtiter plate to measure the absorbance at 600 nm using a Synergy HT microtiter plate reader (BioTek Instruments, Winooski, VT, USA),.

The average OD of three wells for each strain was computed (OD_t_), and the cut-off OD (OD_c_) was determined as being three standard deviations above the average OD of the negative control. The test isolates were classified as non-biofilm forming (N): OD_t_ ≤ OD_c_, weak biofilm forming (W): OD_c_ < OD_t_ ≤ 2 × OD_c_, moderate biofilm forming (M): 2 × OD_c_ < OD_t_ ≤ 4 × OD_c_ and strong biofilm forming isolates (S): OD_t_ > 4 × OD_c_ [26].

### The effect of SCFAs on *E. coli* adhesion

Bacterial adhesion was evaluated by the same technique performed with biofilm production, but instead of incubating the 96-well plates for 24 h, plates were incubated for just 2 h and the procedure was continued as before [27].

### Statistical analysis

GraphPad Prism software was utilized for the statistical analysis employing the one-way ANOVA test (version 7.0, GraphPad Software Inc., La Jolla, CA, USA). Statistics are deemed significant for *P*-values below 0.05. All data were expressed as mean ± SEM.

## Results

### Susceptibility of *E. coli* isolates to β-lactam antibiotic in presence of β-lactamase inhibitors

**Fig. 1:**
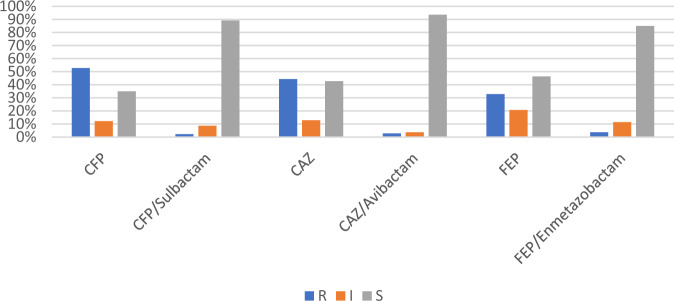
Susceptibility patterns of *E. coli* isolates (*n* = 140) towards different β-Lactams singly and in combination with β-Lactamase inhibitors (CFP Cefoperazone, CAZ Ceftazidime, FEP Cefepime)

Figure 1 shows that cephalosporins were effective against *E. coli* isolates at the following rates: cefoperazone (52.9%), ceftazidime (43.5%), and cefepime (32.1%). The addition of β-lactamase inhibitors restored the susceptibility of the majority of *E. coli* isolates, where 94.3%, 89.2%, 85.7% of the isolates became susceptible to cefoperazone/sulbactam, cefepime/enmetazobactam and ceftazidime/avibactam, respectively. Eighteen *E. coli* isolates remained resistant or had intermediate resistance to β-lactams even after the addition of β-lactamase inhibitors. These isolates were used in the next experiments.

### Susceptibility of resistant *E. coli* isolates to different short chain fatty acids (SCFAs)

Acetic, propionic, and butyric acids showed the same MIC (3750 μg ml^−1^) against all tested isolates. Where, 3750 μg ml^−1^ of SCFAs is approximately equivalent to 60 mM, which is the lowest concentration of SCFAs normally present in human colon.

### Investigating the combined effect of β-lactams/β-lactamase inhibitors along with SCFAs

New combinations that included SCFAs along with cephalosporins and new β-lactamase inhibitors were examined for their therapeutic effect against the eighteen *E. coli* isolates that remained resistant. Table 2 shows the susceptibility of these isolates to each of the three combinations alone and in presence of SCFAs as interpreted according to CLSI guidelines [21].

**Table 2:** Susceptibility of *E. coli* clinical isolates (*n* = 18) towards different β-lactam/β-lactamase inhibitor combinations alone and in combination with ½ MIC of SCFAs

Strain No.	Cefoperazone + Sulbactam	Cefoperazone + Sulbactam + SCFAs	Ceftazidime + Avibactam	Ceftazidime + Avibactam + SCFAs	Cefepime + Enmetazobactam	Cefepime + Enmetazobactam + SCFAs
**1**	32	8	4	2	8	1
**2**	16	2	1	<0.25	4	<0.25
**3**	128	64	16	16	64	8
**10**	32	8	8	<0.25	8	<0.25
**13**	32	0.25	<0.25	<0.25	<0.25	<0.25
**16**	32	8	2	2	8	4
**18**	64	0.5	2	0.5	8	<0.25
**38**	32	<0.25	4	<0.25	8	<0.25
**42**	32	2	1	<0.25	4	<0.25
**48**	32	32	4	2	8	4
**50**	32	2	<0.25	<0.25	2	<0.25
**52**	32	16	4	<0.25	8	4
**56**	128	32	8	4	8	4
**61**	32	16	16	<0.25	16	<0.25
**98**	32	1	2	<0.25	8	<0.25
**102**	8	8	32	2	16	8
**116**	<0.25	<0.25	0.5	<0.25	8	<0.25
**126**	4	4	8	4	4	2

Table 3 shows that the addition of SCFAs enhanced the susceptibility of the tested isolates (*n* = 18) reaching 94.4%, 83.3% and 66.7% for ceftazidime/avibactam, cefoperazone/sulbactam and cefepime/enmetazobactam, respectively. Only one isolate (strain no. 3) remained resistant to cefoperazone/sulbactam and ceftazidime/avibactam after the addition of SCFAs.

**Table 3:** Susceptibility patterns of *E. coli* isolates towards different β-lactam/β-lactamase inhibitor combinations alone and in combination with SCFAs

Combinations	*E. coli* isolates (*n* = 18)					
	Resistant		Intermediate		Sensitive	
	*N*	%	*N*	%	*N*	%
Cefoperazone/Sulbactam	3	16.7%	11	61.1%	4	22.2%
Cefoperazone/Sulbactam + SCFAs	1	5.6%	2	11.1%	15	83.3%
Ceftazidime/Avibactam	3	16.7%	3	16.7%	12	66.6%
Ceftazidime/Avibactam + SCFAs	1	5.6%	–	–	17	94.4%
Cefepime/Enmetazobactam	3	16.7%	13	72.2%	2	11.1%
Cefepime/Enmetazobactam + SCFAs	–	–	6	33.3%	12	66.7%

The addition of SCFAs resulted in a decrease in the MICs of β-lactam/β-lactamase inhibitor combinations. Our results showed that, after the addition of SCFAs to cefoperazone/sulbactam combination, only 3 out of 14 isolates that were resistant to 32 μg ml^−1^ remained resistant, and the number of susceptible isolates (MIC ≤ 16 μg ml^−1^) reached 15 isolates. Furthermore, only one isolate remained resistant to ceftazidime/avibactam combination after the of addition of SCFAs, and the number of isolates having a MIC of less than 0.25 μg ml^−1^ increased from 2 to 10 isolates. When SCFAs were combined with cefepime/enmetazobactam, ten isolates reported MIC values of less than 0.25 μg ml^−1^ and all of the tested isolates were inhibited by MIC ≤ 16 μg ml^−1^ (Table S1).

**Fig. 2:**
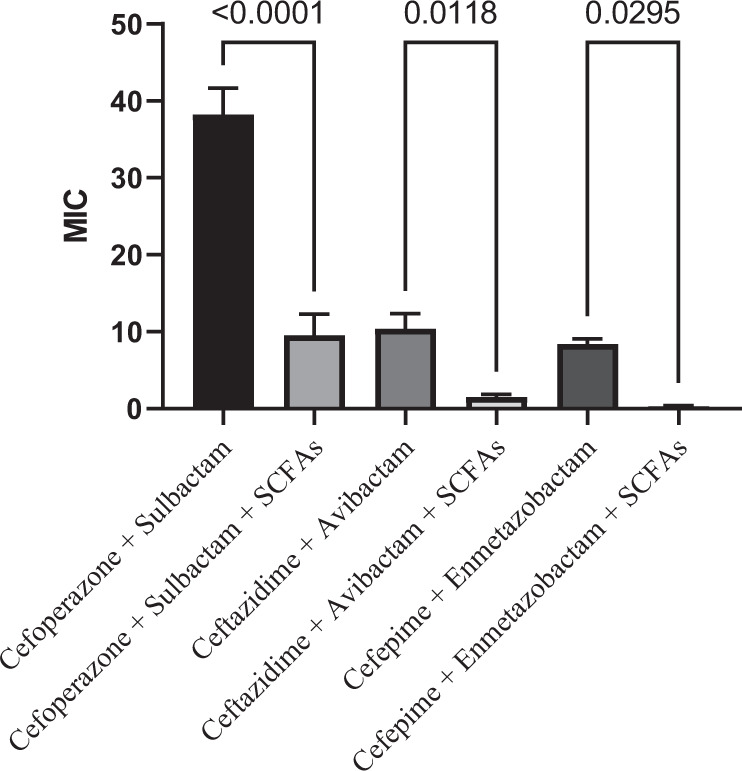
Comparison of average MICs between β-lactam/β-lactamase inhibitor combinations alone and in combination with SCFAs. One way ANOVA test. *P* < 0.05 considered statistically significant. All data were expressed as mean ± SEM

As shown in Fig. 2, and following combination, the MIC values of β**-**lactams dramatically decreased according to statistical analysis, and SCFAs were significantly more effective against most clinical isolates of *E. coli*. The addition of the SCFAs to the three tested combinations significantly enhanced their inhibitory activities with P-values of 0.0001 for cefoperazone/sulbactam, 0.0118 for ceftazidime/avibactam, and 0.0295 for cefepime/enmetazobactam.

### Effect of SCFAs on in vitro growth of *E. coli*

Figure S1 shows the growth curve of a control *E. coli* isolate (without addition of SCFAs) as measured by optical density (OD) at 600 nm. The collinearity between the viable cell number, as CFU, and the optical density was established in a preliminary calibration curve (Fig. S2).

**Fig. 3:**
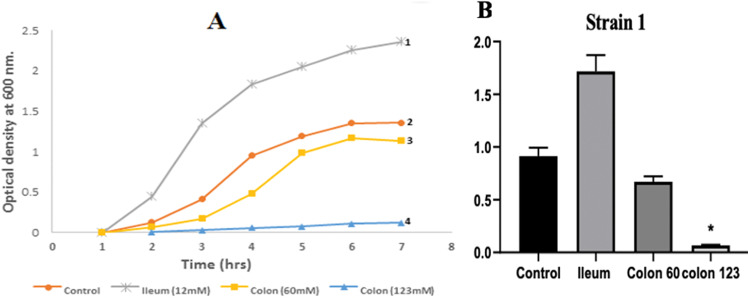
**A** Concentration-dependent effect of SCFAs on growth of representative *E. coli* isolate (no. 1); measured by OD at 600 nm. 1: [12 mM], 2: [Control], 3: [60 mM], 4: [123 mM]. **B** Statistical analysis of the effect of different concentration of SCFAs on *E*. *coli* growth, *P* < 0.05 considered statistically significant. All data were expressed as mean ± SE

A representative of the eighteen combinations-resistant isolates were tested for their growth capacity in the presence of different amounts of SCFAs at different pH [12 mM at pH of 7.4 (ileum conditions), 60 mM and 123 mM, at pH of 6.5 (colon conditions)]. The data represented in Fig. 3 and Table S2 -compared to control- revealed that SCFAs had a great impact on the bacterial growth in a pH-dependent and concentration-dependent manner. Growth curve showed that *E. coli* exhibited increased growth at the low ileal concentration (i-SCFA 12 mM at pH 7.4). On the other hand, high colonic concentration (123 mM c-SCFAs at pH 6.5) significantly suppressed *E. coli* growth, while low colonic concentration (60 m M) had no significant effect on growth.

### The effect of short chain fatty acids on expression of *E. coli* virulence genes

**Fig. 4:**
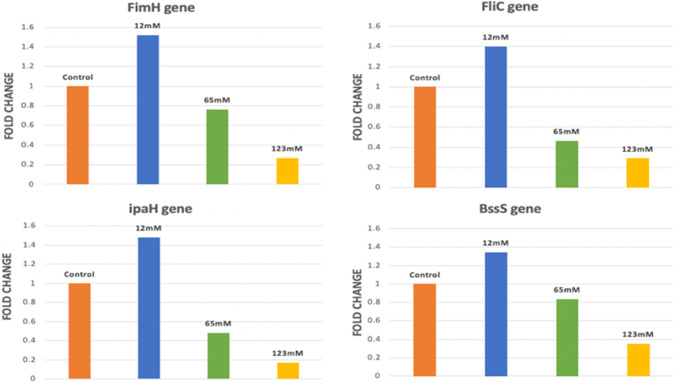
Effect of SCFAs on relative expression levels of virulence genes (*fimH, fliC, ipaH* and *BssS)* in representative *E. coli* isolate (no.1)

The effect of SCFAs on the expression of four virulence genes encoding for; motility (*fliC*), adhesion (*FimH*), invasion (*ipaH*) and biofilm formation (*BssS*) were evaluated. The results showed that SCFAs were able to reduce virulence genes expression in *E. coli* (Table 4, Fig. 4).

**Table 4:** Fold change in virulence genes expression in *E. coli* in response to SCFAs

Condition	12 mM	60 mM	123 mM
***fimH***	1.520979	0.76313	0.267943
***fliC***	1.399586	0.463294	0.291183
***ipaH***	1.479388	0.482968	0.171348
***BssS***	1.342573	0.835088	0.353553

Virulence genes transcription showed that c-SCFAs treatment downregulated all tested virulence genes in a concentration-dependent manner. It was found that *ipaH* and *fliC* genes were down regulated by 0.6-fold after treatment with 60 mM SCFAs, while *BssS* and *FimH* were down regulated by only 0.2 and 0.3-fold; respectively. However, the high-level concentration of SCFAs (123 mM) suppressed the expression of all tested genes, where *BssS* gene expression was decreased by 0.7-fold, followed by *fliC* and *FimH* genes that were suppressed by 0.8-fold, while *ipaH* became the least expressed gene by 0.9-fold.

On the other hand, *E. coli* isolates showed overexpression of all tested genes in presence of i-SCFAs (12 mM at pH 7.4). Where the expression of *FimH* and *ipaH* genes was increased by 0.5 and 0.4-fold; respectively while both *BssS* and *fliC* genes were up regulated by 0.3-fold.

### SCFAs modulate *E. coli* motility

**Fig. 5:**
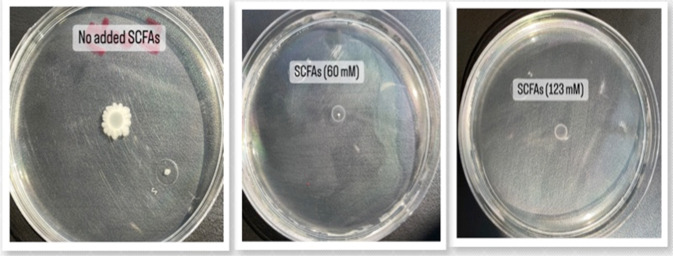
Inhibition of swarming motility of *E. coli* isolate by c-SCFAs

The swarming motility of *E. coli* was evaluated in the presence of c-SCFAs at different concentrations using motility assay test, where agar plates contained different concentrations of SCFAs (Fig. 5). The swarming motility of *E. coli* was completely inhibited at 60 mM and 123 mM c-SCFAs compared to controls.

### The effect of SCFAs on biofilm formation

Biofilm formation of *E. coli* isolates shown in Table 5 revealed that 61% of tested eighteen isolates were strong biofilm forming and 39% of isolates were moderate. In the presence of SCFAs at 60 mM, all the tested isolates were moderate biofilm producers, while at 123 mM all isolates were weak biofilm producers.

**Table 5:** Effects of SCFAs on biofilm formation by clinical *E. coli* isolates

Strain No.	Average OD value		
	Control	SCFAs (60 mM)	SCFAs (123 mM)
**1**	1.243(S)	0.602(M)	0.293(W)
**2**	0.756(M)	0.578(M)	0.321(W)
**3**	1.012(S)	0.623(M)	0.405(W)
**10**	0.874(M)	0.524 (M)	0.374(W)
**13**	0.989(M)	0.531 (M)	0.374(W)
**16**	1.139(S)	0.662 (M)	0.438(W)
**18**	1.111(S)	0.633(M)	0.461(W)
**38**	0.991(M)	0.550(M)	0.388(W)
**42**	1.156(S)	0.731(M)	0.453(W)
**48**	1.021(S)	0.607(M)	0.476(W)
**50**	1.113(S)	0.613(M)	0.483(W)
**52**	0.993(M)	0.580(M)	0.453(W)
**56**	1.048(S)	0.624(M)	0.490(W)
**61**	1.021(S)	0.712(M)	0.365(W)
**98**	1.032(S)	0.576(M)	0.476(W)
**102**	1.121(S)	0.582(M)	0.282(W)
**116**	0.812(M)	0.632(M)	0.425(W)
**126**	0.943(M)	0.702(M)	0.378(W)

*OD* Optical density at 600 nm (Absorbance), *S* strong biofilm (OD > 1), *M* moderate biofilm (OD = 0.51–1), *W* weak biofilm (OD = 0.25–0.5)

### Effect of SCFAs on bacterial adhesion

The result shown in Table 6 revealed that all tested *E. coli* clinical isolates can strongly adhere to polystyrene microtiter plate. However, in the presence of 60 mM of SCFAs all the isolates appeared as moderately adherent. In the presence of 123 mM of SCFAs, around 72% of the isolates were found to be weakly adherent, and 5 out of 18 became non-adherent.

**Table 6:** Effect of SCFAs on *E. coli* adhesion on microtiter plate after 2 h incubation and measuring OD at 600 nm

Strain No.	Average OD value		
	Control	SCFAs (60 mM)	SCFAs (123 mM)
**1**	0.365(S)	0.254(M)	0.085(W)
**2**	0.401(S)	0.194(M)	0.091(W)
**3**	0.332(S)	0.311(M)	0.084(W)
**10**	0.401(S)	0.262(M)	0.081(W)
**13**	0.325(S)	0.173(M)	0.073(N)
**16**	0.524(S)	0.287(M)	0.084(W)
**18**	0.342(S)	0.176(M)	0.065(N)
**38**	0.425(S)	0.283(M)	0.096(W)
**42**	0.358(S)	0.180(M)	0.076(N)
**48**	0.423(S)	0.320(M)	0.087(W)
**50**	0.395(S)	0.205(M)	0.083(W)
**52**	0.462(S)	0.314(M)	0.091(W)
**56**	0.478(S)	0.302(M)	0.098(W)
**61**	0.521(S)	0.192(M)	0.096(W)
**98**	0.451(S)	0.228(M)	0.074(N)
**102**	0.394(S)	0.254(M)	0.102(W)
**116**	0.421(S)	0.190(M)	0.087(W)
**126**	0.516(S)	0.238(M)	0.069(N)

*OD* Optical density at 600 nm (Absorbance). *S* strong adherent (OD > 0.32), *M* moderate adherent (OD = 0.17–0.32), *W* weak adherent (OD = 0.08–0.16), *N* non adherent (OD < 0.08)

## Discussion

β-lactam antibiotics are considered the appropriate choices for treating *E. coli* infections. The inappropriate use that prevails in developing nations have a significant impact on rising rates of resistance and, as a result, rising rates of therapeutic failure [5, 26, 28, 29].

One of the main mechanisms of resistance to β-lactams is the production of β-lactamase enzymes, therefore, β-lactam/β-lactamase inhibitor combinations are particularly valuable treatment options. However, bacterial evolution has progressed and MDR bacteria such as carbapenem-resistant *Enterobacteriaceae* (CRE), are proliferating [30]. In a recent study, it was found that several cephalosporins, notably cefoperazone (52.9%), ceftazidime (43.5%), and cefepime (32.1%), had high rates of resistance when used alone. Furthermore, elevated resistance rates were noticed with older β-lactam/β-lactamase inhibitor combinations such as amoxicillin/clavulanate and ampicillin/sulbactam (40.7% and 42.9%), respectively [14].

Fortunately, the in vitro activity of some β-lactams was potentiated by addition of new β-lactamase inhibitors, for example, the activity of cefoperazone was enhanced by addition of sulbactam. In the present study, it was found that cefoperazone resistance was declined from 52.9% to 2.2%. Similarly, a study by Ku and Yu [31], demonstrated that addition of sulbactam to cefoperazone significantly enhanced the antimicrobial activity against Gram-negative pathogens.

In the present study, high percentage of tested *E. coli* isolates (43.5%) were found to be resistant to ceftazidime. Though, most of our isolates (94.3%) were inhibited by the ceftazidime/avibactam combination. Recently, Yang et al. [32] reported that 78% of their isolates were susceptible to ceftazidime/avibactam. In addition, avibactam-ceftazidime combination had demonstrated high activity against *P. aeruginosa*, and CRE isolates [11].

Furthermore, it was found that enmetazobactam has increased the activity of cefepime, from 43.6% to 85.7%. Similarly, Morrissey et al. [33] reported that enmetazobactam caused a significant reduction in cefepime-MIC_90_, and suggested that this combination might be a valuable option for empirical treatment of serious Gram-negative infections.

SCFAs are of increasing interest for therapeutics development as they have been shown to modulate replication, colonization, and virulence of enteric pathogens [19]. Intestinal SCFAs are by-products of bacterial fermentation. Most microorganisms are susceptible to antimicrobial effects in presence of organic acids [34]. It was proposed that the reason behind their antimicrobial activity, is the ability to diffuse across cell membrane into the bacterial cytoplasm, modifying the intracellular pH and metabolism [35]. It was reported that the concentrations of SCFAs in colon are 10-fold higher than that in ileum, with noticeable differences in luminal pH [36, 37].

Till now, there have been limited studies evaluating the MIC values of SCFAs in food-borne pathogens [38]. In the current study, we investigated the effect of SCFAs with their different concentrations -normally present in colon- on the activity of new β-lactam/β-lactamase inhibitor combinations in addition to their role in growth and virulence of *E. coli* isolated from GIT infections. In this study, the MICs of the three most predominant SCFAs were determined. Acetic, propionic, and butyric acids were found to have the same MIC value (3750 μg ml^−1^) in all tested isolates. This is consistent with a previous study by Lamas et al. [19].

Similarly, another study that evaluated the antimicrobial activity of acetic acid in several pathogens including *E. coli*, found that the MIC were between 1600 μg ml^−1^ and 3100 μg ml^−1^ [39]. Another study showed that organic acids could prevent growth of *E. coli* and *Salmonella* isolates at varying concentrations [40]. In addition, MICs of 15 mg ml^−1^ and 10 mg ml^−1^ of acetic acid were reported against *E. coli* and *Salmonella*; respectively [40].

The present study found that the presence of SCFAs in a concentration that mimic that present normally in the colon, greatly restored the susceptibility of almost *E. coli* isolates against the tested β-lactams, where only one isolate remained resistant. Moreover, MICs values of β-lactam/β-lactamase inhibitor combinations were significantly decreased after the addition of SCFAs; cefoperazone/sulbactam/SCFAs combination (<0.0001) followed by ceftazidime/avibactam/SCFAs (*P-*value of 0.0118) then cefepime/enmetazobactam/SCFAs (*P*-value of 0.0295).

In addition, we examined the effect of SCFAs on the growth and pathogenicity of *E. coli* isolates recovered from GIT infections in the context of alterations in the microenvironment with regard to concentration and pH. The ileal and colonic conditions were modeled using the concentrations and ratios of SCFAs along with pH values. We demonstrated that the concentration and pH of SCFAs had an impact on the growth of *E. coli*, where ileal (i-SCFA) favored growth, and colonic (c-SCFA) suppressed growth. These results are in agreement with that of Zhang et al. [16] who found that under ileal conditions (pH = 7.4; 12 mM), SCFAs significantly potentiated the growth and motility of *E. coli*. However, under colonic conditions (pH = 6.5; 65 to 123 mM), SCFAs significantly inhibited growth in a pH dependent fashion.

We also examined the impact of SCFAs on *E. coli* virulence genes transcription including those encoding for motility (*fliC*), adhesion (*FimH*), invasion (*ipaH*) and biofilm formation (*BssS*) [41, 42].

Overexpression of all tested genes was observed in presence of i-SCFAs (12 mM at pH 7.4). On the other hand, c-SCFA down regulated the virulence genes expression in a concentration-dependent manner. These findings are in agreement with Zhang et al. [43], reporting that under ileal conditions, SCFAs significantly potentiated the growth and motility of *E. coli*. However, in colonic conditions (pH = 6.5; 65–123 mM), SCFAs significantly down-regulated virulence genes expression (*fliC, fimH, htrA, chuA, pks*), especially those linked to adhesion and motility. The adhesin gene *(fimH)* and the motility gene *(fliC)* were both downregulated in 11 out of 15 and 9 out of 15 *E. coli* strains, respectively.

Moreover, our findings were consistent with those reported with *Salmonella* and Enterohemorrhagic *E. coli* (EHEC) isolates. For instance, Lackraj et al. [44] demonstrated that SCFAs simulating the concentration in the small intestine increased the expression of genes involved in EHEC flagella biosynthesis and motility, while doing the opposite for SCFAs simulating the large intestine, where flagella expression by (*FliC*) confirmed a strong, significant 3.7-fold increase under 30 mM SCFA relative to the 172 mM SCFA treatment, when compared to the respective NaCl controls. Similarly, ileal SCFAs increased but colonic SCFAs reduced the expression of *Salmonella* virulence genes that code for invasion of epithelial cells and survival within macrophages [26]. Another study found that, *Salmonella Typhimurium* overexpressed 14 of the genes that were evaluated in the presence of SCFAs as compared to control growth conditions [19].

In summary, significant effort was made in the last ten years to combat the rising resistance rates against classical β-lactams and β-lactam/β-lactamase inhibitor combinations through developing new combinations that target many pathogens including *E. coli*. Our study confirmed that using new combinations restored the susceptibility of most *E. coli* isolates. Moreover, the lowest concentration of SCFAs that is normally produced in colon had significant inhibitory effect on bacterial growth and virulence that could help in restoring the susceptibility of isolates to β-lactam antibiotics in a concentration-dependent manner. To the best of our knowledge, this study is the first to report the effect of SCFAs on therapy by β-lactam/β-lactamase inhibitor combinations. In addition, our results could be promising to control enteric pathogens and warrants more in vivo testing to validate their therapeutic effectiveness.

## Supplementary information

Supplementary Information

## Data Availability

Data will be available upon request.
