# Supplementary material for: Impact of short chain fatty acids (SCFAs) on antimicrobial activity of new β-lactam/β-lactamase inhibitor combinations and on virulence of Escherichia coli isolates
Source: J Antibiot (Tokyo). 2023 Feb 1;76(4):225–35. doi: 10.1038/s41429-023-00595-1 (PMC10040337; doi:10.1038/s41429-023-00595-1)
Supplement: Supplementary file 1 — Supplementary Information [file 41429_2023_595_MOESM1_ESM.docx]

**SUPPLEMNTRY DATA**

**^*^Corresponding Author**

**May A. El-Antrawy**

MSc Degree in Microbiology and Biotechnology,

Department of Microbiology and Immunology, Faculty of Pharmacy, Delta University for Science and Technology, International Coastal Road, Gamasa City, P.O. Box +11152, Mansoura, Dakahlia, Egypt.

**E-mail:** [mayantrawy92@gmail.com](mailto:mayantrawy92@gmail.com)

**Fax**: 002-050-2770145

**Tel**: 002-0112-4222005

**Table (S1): Distribution patterns of MICs of** β**-lactam/** β**-lactamase inhibitor combinations alone and in combination with SCFAs.**

| **MIC range (μg/mL).** | **≤0.25** | **0.5** | **1** | **2** | **4** | **8** | **16** | **32** | **64** | **128** |
| --- | --- | --- | --- | --- | --- | --- | --- | --- | --- | --- |
| **Cefoperazone/Sulbactam** | 1 | - | - | - | 1 | 1 | 1 | 11 | 1 | 2 |
| **Cefoperazone/Sulbactam +SCFAs** | 3 | 1 | 1 | 3 | 1 | 4 | 2 | 2 | 1 | - |
| **Ceftazidime/Avibactam** | 2 | 1 | 2 | 3 | 4 | 3 | 2 | 1 | - | - |
| **Ceftazidime/Avibactam +SCFAs** | 10 | 1 | - | 4 | 2 | - | 1 | - | - | - |
| **Cefepime/Enmetazobactam** | 1 | - | - | 1 | 3 | 10 | 2 | - | 1 | - |
| **Cefepime/Enmetazobactam + SCFAs** | 10 | - | 1 | 1 | 4 | 2 | - | - | - | - |

**Figure (S1): The growth curve of the representative *E. coli* isolate no. 1 at 37°C as measured by optical density (OD) at 600 nm.**

**Figure (S2): Linear correlation between growth of *E. coli* (isolate no. 1) estimated by CFU and absorbance (optical density at 600 nm).Table (S2): The growth of *E. coli* isolates as measured OD at 600 nm before and after addition of SCFAs.**

| **Strain No.** |  | **Experimental conditions** | | | |
| --- | --- | --- | --- | --- | --- |
|  | **Time (hr)** | **Control**  **(No SCFAs)** | **Ileum pH 7.4**  **(12mM)** | **Colon pH 6.5 (60 mM)** | **Colon pH 6.5 (123 mM)** |
| **1** | **2** | 0.126 | 0.451 | 0.071 | 0.010 |
|  | **3** | 0.415 | 1.356 | 0.175 | 0.029 |
|  | **4** | 0.954 | 1.833 | 0.485 | 0.059 |
|  | **5** | 1.195 | 2.052 | 0.986 | 0.078 |
|  | **6** | 1.354 | 2.257 | 1.172 | 0.112 |
|  | **7** | 1.357 | 2.361 | 1.134 | 0.122 |
| **2** | **2** | 0.201 | 0.451 | 0.030 | 0.023 |
|  | **3** | 0.462 | 1.356 | 0.074 | 0.031 |
|  | **4** | 0.987 | 1.833 | 0.197 | 0.055 |
|  | **5** | 1.449 | 2.052 | 0.449 | 0.081 |
|  | **6** | 1.614 | 2.250 | 0.854 | 0.109 |
|  | **7** | 1.668 | 2.354 | 1.080 | 0.130 |
| **3** | **2** | 0.202 | 0.354 | 0.050 | 0.036 |
|  | **3** | 0.651 | 1.178 | 0.136 | 0.054 |
|  | **4** | 1.132 | 1.752 | 0.216 | 0.076 |
|  | **5** | 1.505 | 2.087 | 0.346 | 0.089 |
|  | **6** | 1.624 | 2.291 | 0.552 | 0.101 |
|  | **7** | 1.676 | 2.401 | 0.630 | 0.125 |
| **10** | **2** | 0.220 | 0.421 | 0.090 | 0.032 |
|  | **3** | 0.517 | 1.280 | 0.186 | 0.059 |
|  | **4** | 1.003 | 1.854 | 0.316 | 0.085 |
|  | **5** | 1.410 | 2.101 | 0.482 | 0.090 |
|  | **6** | 1.615 | 2.261 | 0.679 | 0.106 |
|  | **7** | 1.698 | 2.294 | 0.741 | 0.194 |
| **13** | **2** | 0.192 | 0.291 | 0.064 | 0.039 |
|  | **3** | 0.467 | 1.275 | 0.083 | 0.047 |
|  | **4** | 0.904 | 1.724 | 0.209 | 0.060 |
|  | **5** | 1.333 | 1.984 | 0.454 | 0.087 |
|  | **6** | 1.592 | 2.141 | 0.687 | 0.134 |
|  | **7** | 1.625 | 2.252 | 0.751 | 0.168 |
| **16** | **2** | 0.220 | 0.342 | 0.078 | 0.025 |
|  | **3** | 0.568 | 1.154 | 0.133 | 0.034 |
|  | **4** | 0.969 | 1.865 | 0.206 | 0.072 |
|  | **5** | 1.324 | 2.202 | 0.336 | 0.087 |
|  | **6** | 1.558 | 2.351 | 0.513 | 0.119 |
|  | **7** | 1.625 | 2.392 | 0.598 | 0.138 |
| **18** | **2** | 0.170 | 0.301 | 0.056 | 0.030 |
|  | **3** | 0.492 | 1.054 | 0.098 | 0.039 |
|  | **4** | 0.898 | 1.661 | 0.317 | 0.053 |
|  | **5** | 1.273 | 1.975 | 0.524 | 0.083 |
|  | **6** | 1.514 | 2.217 | 0.745 | 0.091 |
|  | **7** | 1.593 | 2.325 | 0.987 | 0.106 |
| **38** | **2** | 0.305 | 0.364 | 0.065 | 0.028 |
|  | **3** | 0.616 | 1.055 | 0.084 | 0.034 |
|  | **4** | 1.001 | 1.714 | 0.212 | 0.037 |
|  | **5** | 1.456 | 2.117 | 0.432 | 0.051 |
|  | **6** | 1.717 | 2.275 | 0.675 | 0.061 |
|  | **7** | 1.774 | 2.284 | 0.751 | 0.086 |
| **42** | **2** | 0.137 | 0.441 | 0.072 | 0.011 |
|  | **3** | 0.395 | 1.255 | 0.775 | 0.028 |
|  | **4** | 0.755 | 1.841 | 0.487 | 0.058 |
|  | **5** | 1.185 | 2.141 | 0.978 | 0.077 |
|  | **6** | 1.454 | 2.296 | 1.165 | 0.114 |
|  | **7** | 1.496 | 2.372 | 1.136 | 0.120 |
| **48** | **2** | 0.221 | 0.327 | 0.031 | 0.024 |
|  | **3** | 0.453 | 1.092 | 0.076 | 0.032 |
|  | **4** | 0.981 | 1.753 | 0.185 | 0.053 |
|  | **5** | 1.469 | 2.033 | 0.448 | 0.082 |
|  | **6** | 1.714 | 2.241 | 0.856 | 0.108 |
|  | **7** | 1.767 | 2.363 | 1.073 | 0.133 |
| **50** | **2** | 0.232 | 0.343 | 0.053 | 0.035 |
|  | **3** | 0.531 | 1.177 | 0.135 | 0.055 |
|  | **4** | 1.003 | 1.765 | 0.218 | 0.078 |
|  | **5** | 1.450 | 2.076 | 0.347 | 0.088 |
|  | **6** | 1.624 | 2.282 | 0.553 | 0.104 |
|  | **7** | 1.695 | 2.412 | 0.632 | 0.127 |
| **52** | **2** | 0.256 | 0.412 | 0.092 | 0.033 |
|  | **3** | 0.517 | 1.119 | 0.187 | 0.058 |
|  | **4** | 0.913 | 1.772 | 0.314 | 0.087 |
|  | **5** | 1.331 | 2.112 | 0.473 | 0.092 |
|  | **6** | 1.515 | 2.253 | 0.665 | 0.108 |
|  | **7** | 1.597 | 2.283 | 0.744 | 0.144 |
| **56** | **2** | 0.193 | 0.280 | 0.066 | 0.038 |
|  | **3** | 0.476 | 1.104 | 0.081 | 0.046 |
|  | **4** | 0.904 | 1.654 | 0.208 | 0.062 |
|  | **5** | 1.333 | 1.973 | 0.455 | 0.086 |
|  | **6** | 1.491 | 2.153 | 0.686 | 0.136 |
|  | **7** | 1.524 | 2.263 | 0.746 | 0.147 |
| **61** | **2** | 0.260 | 0.333 | 0.079 | 0.024 |
|  | **3** | 0.608 | 1.263 | 0.135 | 0.035 |
|  | **4** | 1.096 | 1.876 | 0.217 | 0.075 |
|  | **5** | 1.642 | 2.211 | 0.337 | 0.089 |
|  | **6** | 1.888 | 2.352 | 0.514 | 0.117 |
|  | **7** | 1.974 | 2.385 | 0.596 | 0.139 |
| **98** | **2** | 0.182 | 0.192 | 0.050 | 0.032 |
|  | **3** | 0.462 | 1.043 | 0.136 | 0.038 |
|  | **4** | 0.887 | 1.675 | 0.216 | 0.052 |
|  | **5** | 1.384 | 2.064 | 0.356 | 0.081 |
|  | **6** | 1.713 | 2.226 | 0.562 | 0.095 |
|  | **7** | 1.752 | 2.334 | 0.640 | 0.107 |
| **102** | **2** | 0.315 | 0.455 | 0.078 | 0.027 |
|  | **3** | 0.526 | 1.324 | 0.123 | 0.033 |
|  | **4** | 0.921 | 1.826 | 0.208 | 0.038 |
|  | **5** | 1.345 | 2.126 | 0.346 | 0.056 |
|  | **6** | 1.607 | 2.264 | 0.523 | 0.062 |
|  | **7** | 1.653 | 2.275 | 0.576 | 0.086 |
| **116** | **2** | 0.146 | 0.442 | 0.073 | 0.011 |
|  | **3** | 0.415 | 1.265 | 0.176 | 0.028 |
|  | **4** | 0.844 | 1.844 | 0.486 | 0.057 |
|  | **5** | 1.319 | 2.141 | 0.985 | 0.075 |
|  | **6** | 1.540 | 2.266 | 1.123 | 0.114 |
|  | **7** | 1.596 | 2.372 | 1.133 | 0.124 |
| **126** | **2** | 0.231 | 0.307 | 0.032 | 0.024 |
|  | **3** | 0.543 | 1.188 | 0.073 | 0.033 |
|  | **4** | 1.054 | 1.752 | 0.186 | 0.056 |
|  | **5** | 1.639 | 2.093 | 0.448 | 0.084 |
|  | **6** | 1.914 | 2.261 | 0.854 | 0.107 |
|  | **7** | 1.967 | 2.363 | 1.073 | 0.132 |
